# Effects of ambient temperature on mortality among elderly residents of Chengdu city in Southwest China, 2016–2020: a distributed-lag non-linear time series analysis

**DOI:** 10.1186/s12889-022-14931-x

**Published:** 2023-01-21

**Authors:** Yizhang Xia, Chunli Shi, Yang Li, Xianyan Jiang, Shijuan Ruan, Xufang Gao, Yu Chen, Wei Huang, Mingjiang Li, Rong Xue, Xianying Wen, Xiaojuan Peng, Jianyu Chen, Li Zhang

**Affiliations:** 1 Sichuan Provincial Center for Disease Control and Prevention, No.6, Zhongxue Road, Wuhou District, Chengdu, 610041 China; 2 School of Public Health, Chengdu Medical College, No.783, Xindu Road, Xindu District, Chengdu, 610500 China; 3 Chengdu Center for Disease Control and Prevention, No.6, Longxiang Road, Wuhou District, Chengdu, 610041 China; 4 Zigong Center for Disease Control and Prevention, No.826, Huichuan Road, Ziliujing District, Zigong, 643000 China; 5 Panzhi Hua Center for Disease Control and Prevention, Dong District, No.996, Jichang Road617067, Panzhi Hua, China; 6 Guangyuan Center for Disease Control and Prevention, No.996, Binhebei RoadLizhou District, Guangyuan, 628017 China; 7 Mianyang Center for Disease Control and Prevention, Gaoxin District, No.50, Mianxingdong Road, Mianyang, 621000 China; 8 Yaan Center for Disease Control and Prevention, No.9, Fangcao Road, Yucheng District, Yaan, 625000 China

**Keywords:** Temperature, Mortality, Distributed-lag nonlinear model

## Abstract

**Background:**

With complex changes in the global climate, it is critical to understand how ambient temperature affects health, especially in China. We aimed to assess the effects of temperature on daily mortality, including total non-accidental, cardiovascular disease (CVD), respiratory disease, cerebrovascular disease, and ischemic heart disease (IHD) mortality between 2016 and 2020 in Chengdu, China.

**Methods:**

We obtained daily temperature and mortality data for the period 2016–2020. A Poisson regression model combined with a distributed-lag nonlinear model was used to examine the association between temperature and daily mortality. We investigated the effects of individual characteristics by sex, age, education level, and marital status.

**Results:**

We found significant non-linear effects of temperature on total non-accidental, CVD, respiratory, cerebrovascular, and IHD mortality. Heat effects were immediate and lasted for 0–3 days, whereas cold effects persisted for 7–10 days. The relative risks associated with extreme high temperatures (99th percentile of temperature, 28 °C) over lags of 0–3 days were 1.22 (95% confidence interval [CI]: 1.17, 1.28) for total non-accidental mortality, 1.40 (95% CI: 1.30, 1.50) for CVD morality, 1.34 (95% CI: 1.24, 1.46) for respiratory morality, 1.33 (95% CI: 1.20, 1.47) for cerebrovascular mortality, and 1.38 (95% CI: 1.20, 1.58) for IHD mortality. The relative risks associated with extreme cold temperature (1st percentile of temperature, 3.0 °C) over lags of 0–14 days were 1.32 (95% CI: 1.19, 1.46) for total mortality, 1.45 (95% CI: 1.24, 1.68) for CVD morality, 1.28 (95% CI: 1.09, 1.50) for respiratory morality, 1.36 (95% CI: 1.09, 1.70) for cerebrovascular mortality, and 1.26 (95% CI: 0.95, 1.68) for IHD morality. We found that hot and cold affects were greater in those over 85 years of age, and that women, individuals with low education levels, and those who were widowed, divorced, or never married, were more vulnerable.

**Conclusions:**

This study showed that exposure to hot and cold temperatures in Chengdu was associated with increased mortality, with people over 85 years old, women, those with low education levels, and unmarried individuals being more affected by hot and cold temperatures.

**Supplementary Information:**

The online version contains supplementary material available at 10.1186/s12889-022-14931-x.

## Background

Along with climate change, ambient temperature is a major environmental health risk factor that is considered to be a major public health concern [1–3]. The International Panel on Climate Change predicts that global climate change will result in an increased risk of temperature-related mortality worldwide [4]. Therefore, improved understanding of the relationship between temperature and human health may provide important insights to address the health hazards caused by climate change.

Many studies have shown that high and low temperatures have significant effects on human health [5–8]. For instance, a study in Beijing showed that an increase in the 6-day moving average temperature from moderately hot (30.2 °C) to extremely hot (36.9 °C) resulted in a significant increase in cardiovascular disease (CVD) admissions of 16.1% (95% confidence interval [CI]: 12.8%–28.9%) [9]. Another study in Thessaloniki, Greece reported risk findings for cardiovascular mortality (increase in risk per degree increase in temperature: 4.4%, 95% CI: 2.7%–6.1%) and respiratory mortality (increase in risk per degree increase in temperature: 5.9%, 95% CI: 1.8%–10.3%) at lags of 0–1 days [2]. Additionally, studies suggest that people aged over 65 years are more vulnerable to the health risks of climate change [10].

Current studies regarding the effect of temperature on mortality are mainly from developed countries. As the world's largest developing country and second largest economy, China's rapid economic development has brought with it a series of environmental health problems. However, studies regarding the effect of temperature on mortality in China have only been conducted in a few eastern regions, such as Beijing [11], Tianjin [1], Shanghai [12], and Shenzhen [13], with few studies conducted in western regions, especially in Chengdu. In particular, the effect of low and high temperatures on mortality of people aged over 65 years in Chengdu remains unclear. Therefore, further studies would be urgently needed for a comprehensive understanding of the adverse effects of extreme cold and heat.

In this study, we aimed to determine the impact of extreme temperature on mortality, including total non-accidental, cardiovascular, respiratory, cerebrovascular, and ischemic heart disease (IHD) mortality, in Chengdu, China. We also examined the vulnerable populations by age, gender, education level, and marital status in a stratified analysis. Our findings could provide a new theoretical basis for climate-related public health policies.

## Methods

### Study area and population

**Fig. 1:**
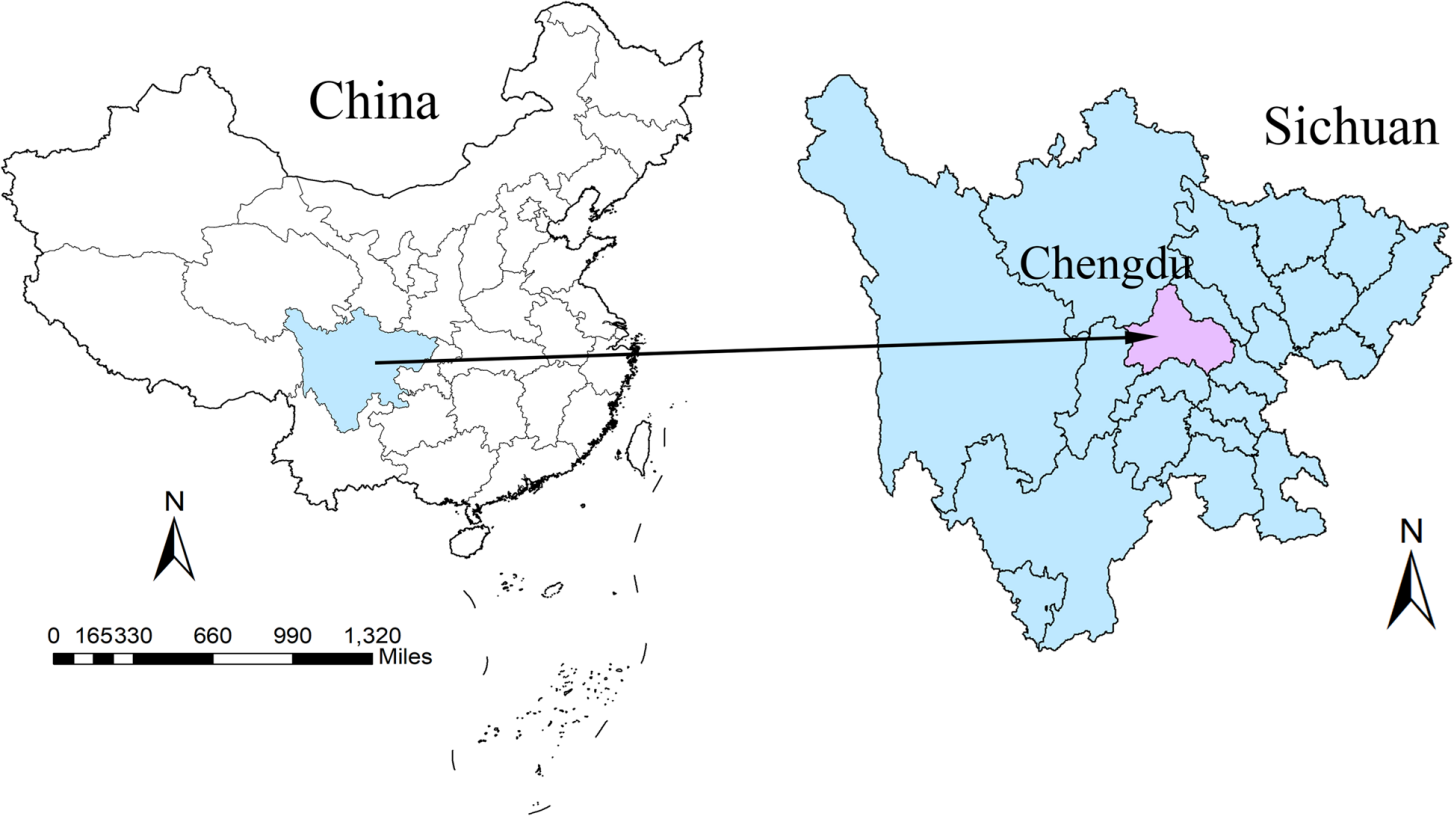
Location of study area in Chengdu city, southwestern China

Chengdu is located in the western portion of the Sichuan Basin and the upstream Yangzi River. Chengdu is the largest city in southwest China, located at 30°05′–31°26′ north latitude and 102°54′–104°53′ east longitude (Fig. **1**). Chengdu has a typical subtropical monsoonal humid and mild climate with four distinct seasons. Chengdu is an important city and economic center in western China. The resident population is over 20 million [14].

## Data collection

Daily non-accidental death counts from January 1, 2016, to December 31, 2020, were obtained from the Population Death Information Registration and Management System (PDIRMS). Mortality data were obtained that covered all mortality-related information for residents of Chengdu city. In the PDIRMS, the death of a resident is confirmed by a hospital or doctor at the resident’s home, and mortality data are subsequently recorded in the system. The causes of death are coded according to the International Classification of Disease, Tenth Revision (ICD-10). The mortality data are classified as death from all causes (A00-R99), respiratory diseases (J00-J99), CVD (I00-I99), cerebrovascular diseases (I60-I69), or IHD (I20-25).

Data on airborne pollutants (particulate matter with diameter 2.5 microns or less [PM_2.5_], particulate matter with diameter 10 microns or less [PM_10_], sulfur dioxide [SO_2_], nitrogen dioxide [NO_2_], and the daily 8-h mean concentrations of ozone [O_3_]) and daily meteorological data (daily maximum, mean, and minimum temperatures [°C] and mean relative humidity [RH%]) were derived from 23 municipal environmental monitoring sites that operated continuously from January 1, 2016, to December 31, 2020, in Chengdu.

## Data analysis

We studied the association between mean ambient temperature and daily counts of total non-accidental mortality, CVD mortality, respiratory mortality, cerebrovascular mortality, and IHD mortality. Several studies have reported that the relationship between temperature and mortality is not linear, but is instead J-, V-, or U-shaped [15–18]. The distribution of temperature effects over days or weeks after exposure is often dealt with by establishing distributed-lag models [19]. Thus, we used distributed-lag non-linear models (DLNM) to estimate the non-linear and lag effects of temperature on daily mortality [15, 20, 21]. Long-term trends, relative humidity and ambient air pollutants (PM_2.5_, O_3_) were controlled in the model as potential confounders. A Poisson regression with a quasi-Poisson function for the daily counts of deaths was constructed, which was specified as follows:

$$Log[E(Yt)] = \alpha + cb(Tempt,l)+ns(PM2.5t,l,3) + ns(O3t,l,3) + ns(Relative Humidityt,l,3) + ns(Time,8*5) + DOWt,$$

where *Yt* is the observed number of daily deaths at day *t*; α is the intercept; *cb*(*Temp*_*t,l*_) is the cross-basis matrix produced by DLNM to model the non-linear and distributed lag effects of ambient temperature from the current day (lag0) to the fourteenth day (lag14); *ns*(*PM*_2.5t*,l*_*,*3) is a natural cubic spline of PM_2.5_ with three degrees of freedom (*df*); *ns*(*O*_3*t,l*_*,*3) is a natural cubic spline of O_3_ with three df; *ns*(*RH*_*t,l*_*,*3) is a natural cubic spline of relative humidity with three df; and ns(*Time*,8*5) represents natural spline function with eight *df* per year to control long-term and seasonal trends [22]. *DOW*_*t*_ is used to control the effect of day of week. We calculated the relative risk (RR) of mortality by comparing extreme cold (1st percentile of temperature) and extreme hot (99th percentile of temperature) temperatures with the minimum mortality temperature. Akaike's Information Criterion for quasi-Poisson models was used to selected the temperature threshold and *df* for temperature and lag [23]. Additionally, we stratified the analysis by gender, age group, education level, and marital status through the above steps.

We tested the statistical significance of differences between effect estimates of the strata of a potential effect modifier (e.g., the difference between low education and high education) by calculating the 95% confidence interval (CI). The equation was as follows:

$$\left({\widehat{Q}}_{1}-{\widehat{Q}}_{2}\right)\pm 1.96\sqrt{{\left(S{\widehat{E}}_{1}\right)}^{2}+{\left(S{\widehat{E}}_{2}\right)}^{2}}$$

where ${\widehat{Q}}_{1}$ and ${\widehat{Q}}_{2}$ are the estimates for the two categories, and ${\left(S{\widehat{E}}_{1}\right)}^{2}$ and ${\left(S{\widehat{E}}_{2}\right)}^{2}$ are their respective standard errors [24].

We used sensitive analyses to assess stability of the model by changing the *df* of the model, including for long-term trends (*df* = 6–9), major air pollutants, and relative humidity (*df* = 3–5). All statistical tests were two-sided, and values of p < 0.05 were considered statistically significant. All analyses were performed using R software version 4.1.2, and the “dlnm” package was used to create the DLNM.

## Results

During the study period (January 1, 2016, to December 31, 2020), there were 343,313 non-accidental deaths in total; 116,661 individuals (33.9%) died from CVD; 98,762 (28.8%) died from respiratory disease; 52,533 (15.3%) died from cerebrovascular disease, and 27,464 individuals (8.0%) died from IHD. The daily average number of deaths due to total non-accidental, CVD, respiratory, cerebrovascular and IHD mortality was 188, 64, 54, 29, and 15, respectively. Daily mean temperature and relative humidity were 16.8 °C (range: − 1.6 °C–30.1 °C) and 79.9% (range: 36.0%–99.0%), respectively. Daily mean concentrations of air pollutants are shown in Table 1.

**Table 1:** Summary statistics of daily cause-specific mortality, air pollutants, and weather conditions in Chengdu, 2016–2020 (1827 days)

Variables	Mean	SD	Min	1st	20%	50%	90%	99%	Max
Total (non-accidental)	187.9	39.3	106.0	124.0	156.0	180.0	246.0	303.0	340.0
CVD	63.9	16.1	30.0	35.0	50.0	62.0	86.0	108.0	119.0
Respiratory	54.1	19.0	20.0	27.0	39.0	49.0	79.0	119.0	136.0
Cerebrovascular	28.8	7.8	10.0	14.0	22.0	28.0	40.0	48.0	65.0
IHD	15.0	5.3	3.0	5.0	10.0	14.0	22.0	30.0	39.0
Air pollutants									
PM_2.5_ (µg/m3)	49.5	34.7	3.0	8.1	22.4	39.6	95.0	170.1	259.8
PM_10_ (µg/m3)	78.5	49.9	5.5	15.3	38.7	66.6	143.1	247.0	358.1
SO_2_ (µg/m3)	10.0	4.8	3.4	4.0	5.8	8.6	16.6	24.8	29.8
NO_2_ (µg/m3)	38.0	13.4	7.0	13.8	26.3	36.2	56.5	73.4	91.1
O_3_ (µg/m3)	95.1	49.4	10.3	17.8	51.7	84.4	166.3	219.4	278.0
Meteorological variables									
Mean temperature (°C)	16.8	7.4	-1.6	3.0	8.6	17.1	26.3	29.0	27.0
Minimum temperature (°C)	13.5	7.2	-6.2	-1.4	6.1	14.3	23.0	24.7	37.2
Maximum temperature (°C)	79.9	8.2	3.3	6.1	12.8	21.7	32.0	35.6	99.0
Relative humidity (%)		9.8	36.0	52.0	72.0	81.0	92.0	97.0	

^*^Min minimum, *Max* maximum, *SD* standard deviation

Table 2 shows the Spearman’s correlation coefficients of air pollutants and meteorological conditions. The results showed correlations between air pollutants and meteorological conditions, except between mean temperature and SO_2_, and between mean temperature and relative humidity.

**Table 2:** Spearman’s correlation coefficients of weather conditions and air pollutants in Chengdu, China (2016–2020)

Variables	PM_2.5_	O_3_	SO_2_	NO_2_	Mean temperature	Relative humidity
PM_2.5_	1					
O_3_	− 0.20*	1				
SO_2_	0.61*	0.16*	1			
NO_2_	0.80*	− 0.17*	0.65*	1		
Mean temperature	− 0.51*	0.72*	− 0.04	− 0.41*	1	
Relative humidity	− 0.13*	− 0.49*	− 0.21*	− 0.16*	− 0.04	1

^*^p < 0.05 for all correlation coefficients

**Fig. 2:**
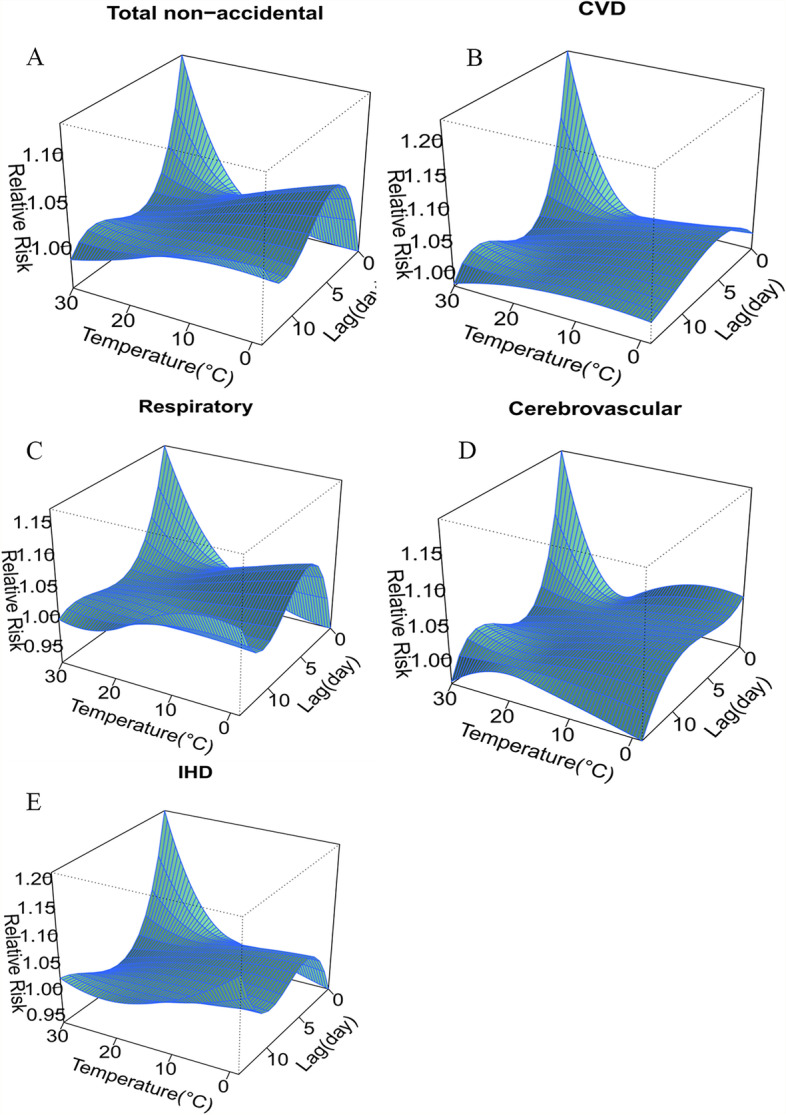
Relative risks of cause-specific mortality by mean temperature (°C) and lag in Chengdu, China, during 2016–2020. A–E

The three-dimensional plots revealed higher relative risks at hot and cold temperatures after fortnight lag. Specifically, a non-linear relationship was found between mean temperature and total non-accidental, CVD, respiratory, cerebrovascular and IHD deaths (Fig. 2 A–E).

**Fig. 3:**
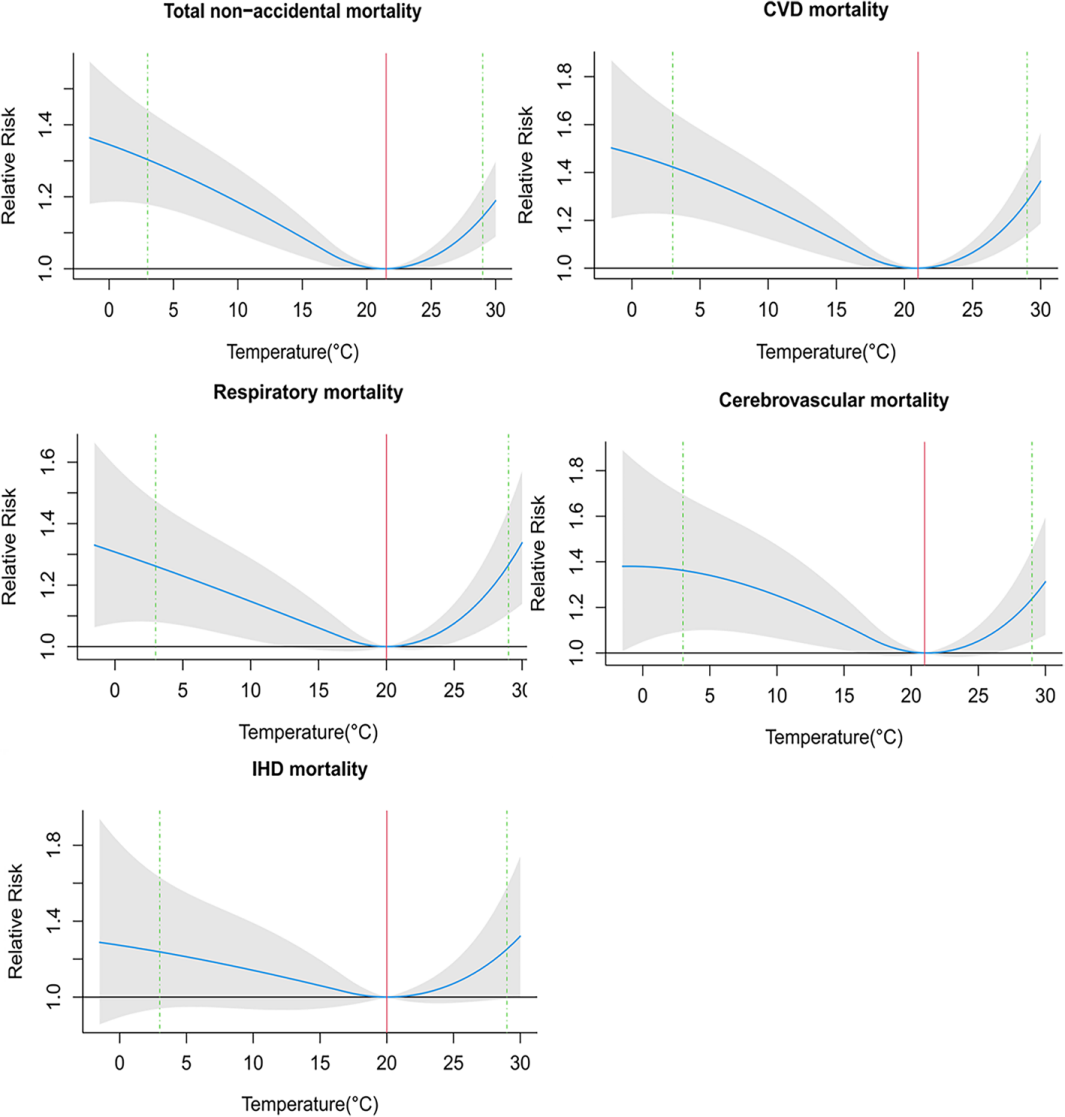
Estimated relative risks of mean temperature (°C) over lags 0–14 days on total non-accidental, CVD, respiratory, cerebrovascular, and IHD mortality. Blue lines are mean relative risks and gray areas are 95% CIs of risk estimates

Figure 3 shows the estimated cumulative effects of mean temperature on total non-accidental, CVD, respiratory, cerebrovascular, and IHD mortality at lags of 0–14 days. The relationships of mean temperature with total non-accidental, CVD, respiratory, cerebrovascular, and IHD mortality were non-linear, with higher relative risks at cold and hot temperatures. The temperature with the minimum mortality risks for total non-accidental, CVD, respiratory, cerebrovascular, and IHD were 21.5 °C, 21 °C, 20 °C, 21 °C, and 20.5 °C, respectively.

**Fig. 4:**
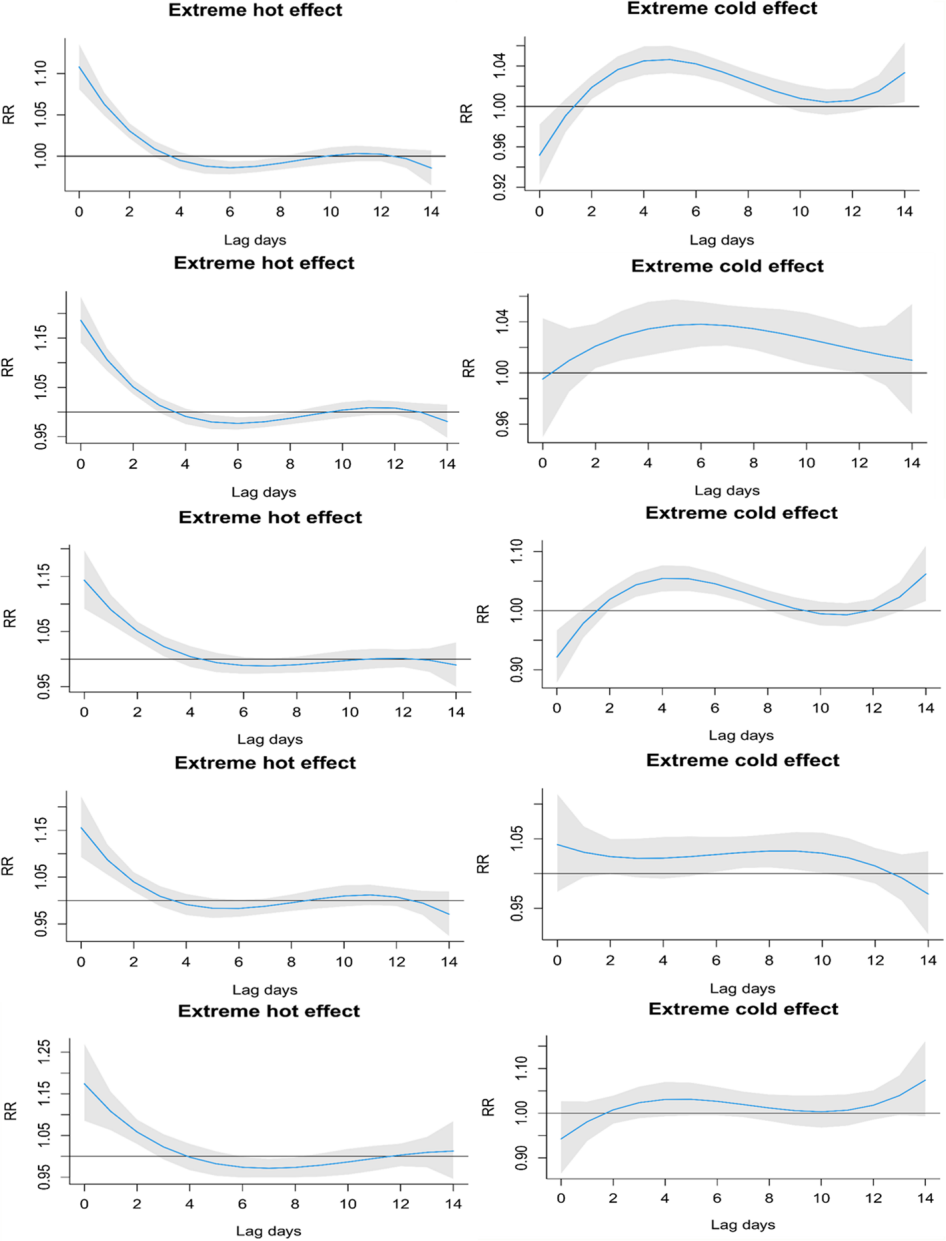
Estimated relative risks of extreme cold and extreme hot temperatures over lags 0–14 days on total non-accidental, CVD, respiratory, cerebrovascular, and IHD mortality. Extreme cold temperature: 1st percentile of temperature, 3.0 °C; extreme hot temperature: 99th percentile of temperature, 29.0 °C

Figure 4 shows the estimated risks of daily mortality associated with extreme cold (3 °C) and extreme hot temperatures (29 °C) at lags of 0–14 days. Significant effects of extreme cold temperatures were observed after 0–2 days and persisted for 7–10 days. The most significant and strongest effects of extreme hot temperatures were observed on the current day and lasted for only 0–3 days.

We calculated the overall effects of mean temperature on total non-accidental, CVD, cerebrovascular, respiratory, and IHD mortality for lags of 0–1, 0–3, 0–7, and 0–14 days (**Table ****3**). The results showed that the relative risks associated with extreme cold temperature (1st percentile of temperature, 3.0 °C) over lags 0–14 days were 1.32 (95% CI: 1.19, 1.46) for total mortality, 1.45 (95% CI: 1.24, 1.68) for CVD morality, 1.28 (95% CI: 1.09, 1.50) for respiratory morality, 1.36 (95% CI: 1.09, 1.70) for cerebrovascular mortality, and 1.26 (95% CI: 0.95, 1.68) for IHD morality, respectively. The relative risks associated with extreme hot temperature (99th percentile of temperature, 29 °C) over lags 0–3 days were 1.22 (95% CI: 1.17, 1.28) for total mortality, 1.40 (95% CI: 1.30, 1.50) for CVD morality, 1.34 (95% CI: 1.24, 1.46) for respiratory morality, 1.33 (95% CI: 1.20, 1.47) for cerebrovascular mortality, and 1.38 (95% CI: 1.20, 1.58) for IHD mortality.

**Table 3:** Cumulative relative risks of extreme hot and extreme cold temperatures on daily mortality, compared with the minimum mortality temperature

	Lag	Total mortality	Cardiovascular mortality	Respiratory mortality	Cerebrovascular mortality	IHD mortality
Extreme hot	0–1	1.18 (1.13,1.22)*	1.31 (1.24,1.39)*	1.25 (1.17,1.34)*	1.26 (1.16,1.38)*	1.28 (1.14,1.44)*
	0–3	1.22 (1.17,1.28)*	1.40 (1.30,1.50)*	1.34 (1.24,1.46)*	1.33 (1.20,1.47)*	1.38 (1.20,1.58)*
	0–7	1.17 (1.11,1.23)*	1.30 (1.20,1.41)*	1.30 (1.19,1.43)*	1.26 (1.12,1.41)*	1.29 (1.10,1.15)*
	0–14	1.14 (1.06,1.23)*	1.28 (1.14,1.43)*	1.27 (1.11,1.45)*	1.25 (1.06,1.47)*	1.24 (0.98,1.56)
Extreme cold	0–1	0.94 (0.90,0.99)	1.01 (0.94,1.08)	0.91 (0.84,0.97)	1.07 (0.96,1.18)	0.93 (0.82,1.06)
	0–3	1.00 (0.94,1.06)	1.06 (0.97,1.15)	0.96 (0.88,1.05)	1.11 (0.99,1.26)	0.97 (0.83,1.13)
	0–7	1.18 (1.10,1.26)*	1.23 (1.11,1.36)*	1.16 (1.04,1.29)*	1.23 (1.06,1.43)*	1.09 (0.90,1.31)
	0–14	1.32 (1.19,1.46)*	1.45 (1.24,1.68)*	1.28 (1.09,1.50)*	1.36 (1.09,1.70)*	1.26 (0.95,1.68)

Extreme cold temperature: 1st percentile of temperature, 3 °C; extreme hot temperature: 99th percentile of temperature, 29 °C; minimum mortality temperature for total non-accidental, cardiovascular, respiratory, cerebrovascular, and ischemic heart disease mortality: 21.5 °C, 21 °C, 20 °C, 21 °C, and 20.5 °C, respectively

^*^ *p* < 0.05

**Fig. 5:**
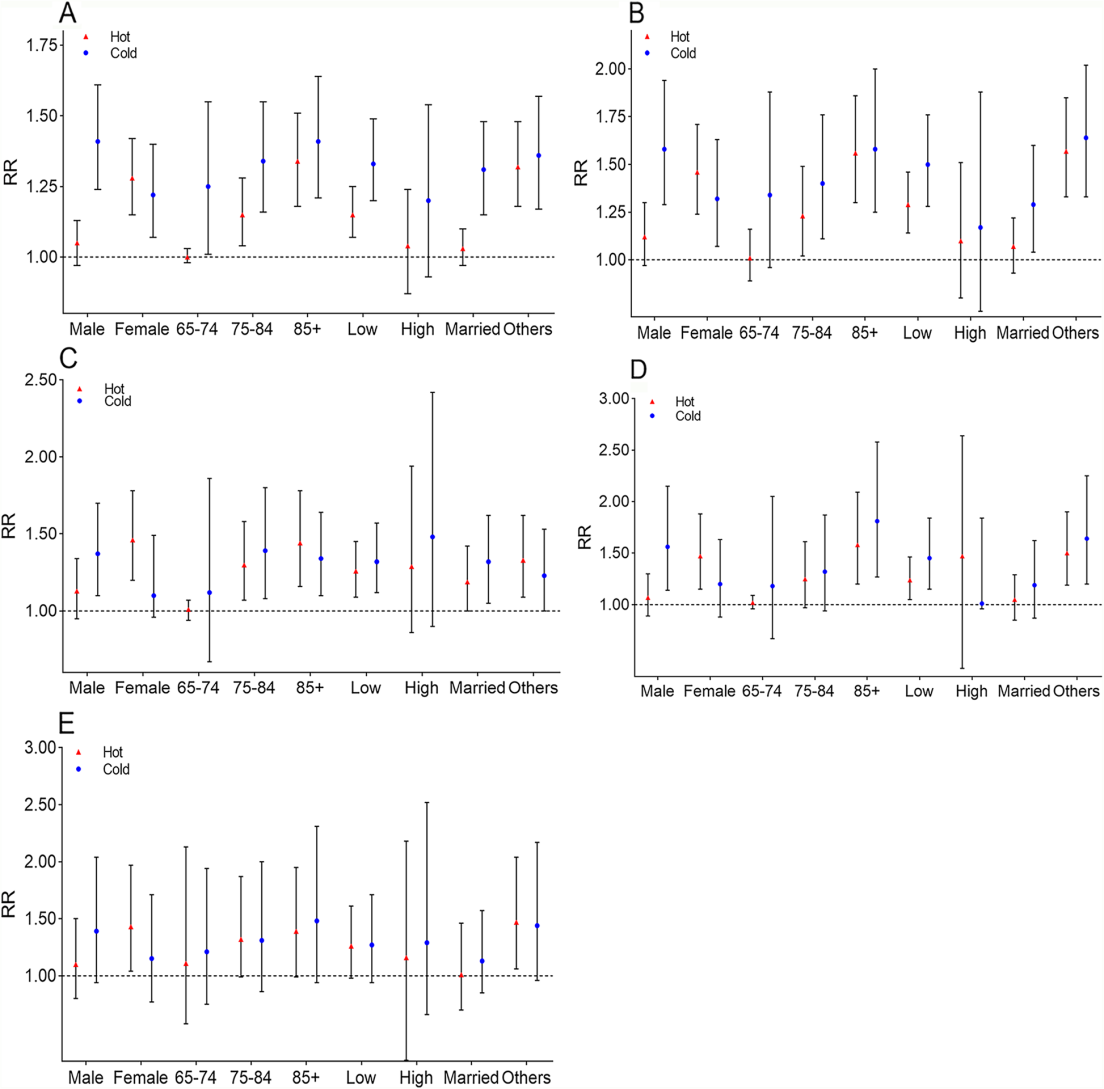
Relative risk (RR) of cold and heat on mortality associated with different diseases among older adults in Chengdu, China. A–E) total non-accidental, cardiovascular, respiratory, cerebrovascular, and ischemic heart disease-related deaths. Low education level: illiterate and primary; high education level: junior high school and above. Other marital status: widowed, divorced, and never married

Figure 5 presents the relative risks of low and high temperatures for disease-related mortality in subgroups by sex, age, education level, and marital status. The results indicated that the relationship between temperature and mortality was more pronounced among adults over 85 years of age, women, individuals with low education, and those with other marital status.

## Discussion

In the present study, we examined the effects of ambient temperature on cause-specific mortality among older adults in Chengdu, China, during the years 2016–2020. The results showed that both cold and high temperatures increased the risk of mortality. J-shaped and U-shaped relationships were found between temperature and all etiology-specific mortality categories. The relationship between temperature and mortality was more pronounced among adults over 85 years of age, women, individuals with low education, and those who were divorced, which is consistent with previous studies [19, 25, 26]. Some prior research reported a significantly stronger association between temperature and disease-related mortality in women than in men [27, 28], while some reported the opposite [29]. Furthermore, our results showed that the effects of cold temperatures on mortality were much larger than those of hot temperatures, with the effects of cold temperatures occurring after 0–2 days and lasting 7–10 days, whereas the effects of high temperatures were short-lived. Similar results have been found in previous studies in Beijing [30], Guangzhou [31], Hong Kong [32], and Thailand [33].

Many studies have confirmed that high and low temperatures lead to an increased risk of death, that people in different regions are adapted to different climates, and that the degree of influence of temperature varies according to geography, population, and environmental conditions [8, 16, 34]. Our study confirmed the findings of other studies. It is worth noting that in comparing the 99th percentile of temperature (29 °C) to the minimum mortality temperature (21.5 °C), we found that exposure to 2-day average high temperatures increased non-accidental overall mortality by 18% (95% CI: 13%–22%), which is greater than that reported in a study conducted in Jinan (3.8% 95% CI: 2.6%–5.0%) [35]. However, a study in Wuhan found a similar heat effect, with a 17.7% (12.6%–22.9%) increase in total non-accidental mortality at 99% temperature (34.7 °C) relative to the minimum mortality temperature (31.7 °C) [36]. Consistent with the results of the study in Wuhan [30], we did not find a significant effect of low temperature on IHD mortality, possibly because the relatively small number of deaths owing to IHD limited our ability to detect an association between temperature and IHD.

Many previous studies have demonstrated that older adults are more sensitive to the effects of temperature [35, 37, 38]. Our research indicated that older adults are more susceptible to the effects of cold temperatures. This is consistent with the findings of a study in Kuwait [39], where the incidence of death owing to CVD was higher in old people, especially during cold periods. Several studies have reported that sensitivity to heat and cold exposure may vary among populations in different regions owing to adaptation to local climate [5, 8, 40]. Population age structure, socioeconomic conditions, education, health care, infrastructure development, housing quality, and air conditioning use also influence the effects of hot and cold temperatures on human health [8, 41, 42]. For example, an Australian study found a diminished association between temperature and cardiovascular mortality in people over the age of 85 years who lived in areas of higher socioeconomic status [43].

When analyzing the effect of temperature on mortality according to cause of death, we found that the effects of hot and cold temperatures on CVD-related mortality were all greater than those of non-accidental death and mortality related to respiratory diseases, cerebrovascular diseases, and IHD, which is consistent with the results of a study in Suzhou, China [19]. CVD was the leading cause of death among elderly residents of Chengdu, accounting for 33.9% of all registered deaths during the study period. In addition, previous studies have confirmed that the effect of temperature on mortality owing to different diseases is not consistent. For example, many studies have found that the effects of heat and cold was more pronounced in CVD mortality. However, in a study in Jinan, China, low temperature was found to significantly increase mortality from respiratory diseases; at high temperatures, there was an increase in deaths from respiratory diseases, but the effect was not statistically significant [44]. Additionally, a study in Chiang Mai, Thailand, showed that high temperatures significantly increased mortality from respiratory diseases [45].

Our findings suggested that people over 85 years of age are more sensitive to temperature and have lower mortality rates in the age group 65–74 years, showing more sensitivity to temperature with older age, which confirms previous findings by Breitner [25]. As mentioned in the published literature, concomitant diseases and weakened thermoregulatory mechanisms among older adults may be important factors contributing to mortality, and older people with chronic diseases, such as cardiovascular or respiratory diseases, are usually more vulnerable to hot weather than other groups [46, 47]. Another important finding was that the cold effect was greater than the heat effect for all disease mortality in those aged 85 years and older, except for the cold effect on respiratory disease mortality, which was slightly smaller than the heat effect. In China, the number of people aged 65 years and older reached 190 million in 2020, accounting for 13.5% of the total population. It is expected that by 2050, the proportion of people aged 65 years and above will exceed 25%. China's disabled elderly population will also exceed 97.5 million by approximately 2050. For disabled older people, the inability to take effective measures when they are in hot or cold conditions can lead to an increased risk of death. Considering that older people are more sensitive to low temperatures and given the rapid aging of China's population, the adverse effects of low temperatures on older adults require attention. In addition, tailored measures of treatment care can substantially improve the health and wellbeing of older populations.

We found significantly greater heat effects in women than in men, and greater cold effects in men than in women, although the differences were not significant. This is similar to the results of a previous study conducted in Barcelona, Spain [26]. There are also some differences in previous studies regarding correction owing to the effect of sex on mortality. Some studies have shown a significantly stronger association between temperature and disease-related mortality in women than in men [27, 28] whereas others have found a greater risk in men [29]. In contrast, several other studies have found no sex differences; differences in temperature effects between women and men were influenced by the study site and study population [19, 48]. For example, the reported risk of heat effects on total mortality was greater among men in Shanghai, China, and among women of all ages in Fukuoka, Japan [49, 50].

Another conclusion that can be drawn from our results is that older Chengdu residents with low educational attainment are more susceptible to temperature-related mortality, with both hot and cold effects being significant. Past studies have also confirmed that people with lower education levels are more susceptible to temperature-related mortality. For example, Yang et al. [51] found that the effect of heat and cold was greater for those with lower levels of education. Li et al. [35] reported that low levels of education were associated with heat-related mortality. Wang et al. [19] did not observe a significant modifying effect of education level, although they reported a greater vulnerability of residents with low levels of education to temperature-related mortality. One explanation for this is that people with low levels of education may be more exposed to cold or heat and may have poor living and housing conditions, limited access to health care, and may lack knowledge about precautions against cold and heat exposure, which may contribute to their increased risk of death [52, 53].

Another important finding is that marital status can also explain the association between temperature and mortality. We divided marital status into married and other (widowed, divorced, and never married). We found that non-married people had higher mortality associated with heat and cold than married people, although the difference in the cold effect was not significant. This is consistent with results of a study conducted in São Paulo, Brazil, showing that widowed individuals had higher mortality associated with cold and heat [54]. Many studies have reported that marriage has a "protective effect" on health [55–58]. We speculate that married people are better off financially and can share health benefits with their spouse; married couples may also be more emotionally supportive of each other and have a more positive and optimistic attitude toward life.

In a hot environment, increased body temperature redistributes blood flow to the skin, and the body releases heat through mechanisms such as skin sweat secretion and vasodilation, which can lead to a loss of salt and water from the body. If not adequately replenished, this can lead to dehydration, which decreases blood volume and can ultimately increase cardiovascular strain [7]. Past evidence suggests that the longer a heat wave lasts, the greater the risk of CVD death. Older adults, women, and outdoor workers have higher rates of death [59]. Notably, when the body's thermoregulatory capacity is diminished and the internal temperature is too high (39 °C–40 °C), kidney and liver damage, as well as central nervous system damage, can occur [7].

In contrast, when in a cold environment, skin temperature decreases and vascular tone decreases, causing the body to increase metabolic heat production and begin to shiver to maintain the body's core temperature [60]. However, it has been shown that an inability to maintain core temperature in severely cold environments among people over 65 years of age can lead to tissue damage [61].

The robustness of our model was tested using sensitivity analysis. For temporal trends in the model from 6 to 9, airborne pollutants and relative humidity from 3 to 5 were included (Tables S1 and S2). The RRs calculated according to different *df* were similar. Therefore, the results calculated by the model are reliable.

To our knowledge, this was the first study to explore the association of multiple indicators for temperature with mortality from four specific diseases in the central Sichuan basin of southwest China. Based on the advantages of the PDIRMS, we used realistic and reliable mortality data for the entire city. With current global warming and the frequent occurrence of extreme weather events, the present findings may be critical in reducing temperature-related mortality and provide a theoretical basis for follow-up research. However, this study also has some limitations. First, the study was conducted in the central Sichuan basin, and the data were from only one city, which makes it difficult to generalize our results to other regions considering geographic characteristics, meteorological conditions, and other uncontrollable factors. Second, data for cause of death were classified according to the ICD-10 code on the death certificate, which may be biased. Third, pollutant and meteorological data were from fixed municipal environmental monitoring sites rather than individual exposures, which may result in some unavoidable assessment errors. Further research for evaluating the overall situation in multiple cities is needed to more accurately analyze the effects of ambient temperature on mortality among elderly residents. Additionally, the results would be more reliable if the confounding of mortality risk factors could be excluded in future related studies.

## Conclusions

The results of this study showed that exposure to hot and cold temperatures in Chengdu was associated with increased mortality, with people over 85 years old, women, those with low education levels, and unmarried individuals being more affected by hot and cold temperatures.

## Supplementary Information

**Additional file 1: Table S1. **Time trend freedom 6-9** Table S2. **Air pollutants and relative humidity trend freedom 3-5.

## Data Availability

The datasets generated and/or analysed during the current study are not publicly available due involvement of detailed information on regional population deaths, which needs to be kept confidential but are available from the corresponding author on reasonable request.

## Abbreviations

PM2.5: Particulate matter < 2.5 μm in aerodynamic diameter
SO2: Sulfur dioxide
NO2: Nitrogen dioxide
CO: Carbon monoxide
O3-8 h: Daily 8-h mean concentrations of ozone
RH: Relative humidity
MMT: Minimum mortality temperature
DLNM: Distributed-lag non-linear models
CVD: Cardiovascular disease
IHD: Ischemic heart disease
SD: Standard deviation
RR: Relative risk
CI: Confidence interval

## References

[1] Guo Y, Barnett AG, Pan X, Yu W, Tong S (2011). The impact of temperature on mortality in Tianjin, China: a case-crossover design with a distributed lag nonlinear model. Environ Health Perspect.

[2] Kouis P, Kakkoura M, Ziogas K, Paschalidou AΚ, Papatheodorou SI (2019). The effect of ambient air temperature on cardiovascular and respiratory mortality in Thessaloniki. Greece Sci Total Environ.

[3] Lee H, Myung W, Kim H, Lee E-M, Kim H (2020). Association between ambient temperature and injury by intentions and mechanisms: A case-crossover design with a distributed lag nonlinear model. Sci Total Environ.

[4] Change, Climate I: "The physical science basis." Contribution of working group I to the fifth assessment report of the intergovernmental panel on climate change. 2013.

[5] Ha J, Shin Y, Kim H (2011). Distributed lag effects in the relationship between temperature and mortality in three major cities in South Korea. Sci Total Environ.

[6] Rodrigues M, Santana P, Rocha A (2019). Effects of extreme temperatures on cerebrovascular mortality in Lisbon: a distributed lag non-linear model. Int J Biometeorol.

[7] Kristie L, Ebi AC (2021). Peter Berry, Carolyn Broderick, Richard de Dear, George Havenith, Yasushi Honda, R Sari Kovats, Wei Ma, Arunima Malik, Nathan B Morris, Lars Nybo, Sonia I Seneviratne, Jennifer Vanos, Ollie Jay: Hot weather and heat extremes health risks. The Lancet.

[8] Silveira IH, Oliveira BFA, Cortes TR, Junger WL (2019). The effect of ambient temperature on cardiovascular mortality in 27 Brazilian cities. Sci Total Environ.

[9] Aklilu D, Wang T, Amsalu E, Feng W, Li Z, Li X, Tao L, Luo Y, Guo M, Liu X (2020). Short-term effects of extreme temperatures on cause specific cardiovascular admissions in Beijing. China Environ Res.

[10] Cai W, Zhang C, Zhang S, Bai Y, Callaghan M, Chang N, Chen B, Chen H, Cheng L, Cui X (2022). The 2022 China report of the Lancet Countdown on health and climate change: leveraging climate actions for healthy ageing. Lancet Public Health.

[11] Zhaoxing Tian SL (2012). JZ, JJKJaYG: Ambient temperature and coronary heart disease mortality in Beijing, China a time series study. Environ Health Perspect.

[12] Yang C, Meng X, Chen R, Cai J, Zhao Z, Wan Y, Kan H (2015). Long-term variations in the association between ambient temperature and daily cardiovascular mortality in Shanghai. China Sci Total Environ.

[13] Lian T, Fu Y, Sun M, Yin M, Zhang Y, Huang L, Huang J, Xu Z, Mao C, Ni J (2020). Effect of temperature on accidental human mortality: A time-series analysis in Shenzhen, Guangdong Province in China. Sci Rep.

[14] Resident Population (Year-end) by region. Sichuan Statistical Yearbook [http://tjj.sc.gov.cn/scstjj/c105855/nj.shtml]

[15] Gasparrini A, Armstrong B, Kenward MG (2010). Distributed lag non-linear models. Stat Med.

[16] Chung JY, Honda Y, Hong YC, Pan XC, Guo YL, Kim H (2009). Ambient temperature and mortality: an international study in four capital cities of East Asia. Sci Total Environ.

[17] McMichael AJ, Wilkinson P, Kovats RS, Pattenden S, Hajat S, Armstrong B, Vajanapoom N, Niciu EM, Mahomed H, Kingkeow C (2008). International study of temperature, heat and urban mortality: the 'ISOTHURM' project. Int J Epidemiol.

[18] Medina-Ramon M, Schwartz J (2007). Temperature, temperature extremes, and mortality: a study of acclimatisation and effect modification in 50 US cities. Occup Environ Med.

[19] Wang C, Chen R, Kuang X, Duan X, Kan H (2014). Temperature and daily mortality in Suzhou, China: a time series analysis. Sci Total Environ.

[20] Armstrong B (2006). Models for the relationship between ambient temperature and daily mortality. Epidemiology.

[21] Gasparrini A (2011). Distributed lag linear and non-linear models in R the package dlnm. J Stat Softw.

[22] Achebak H, Devolder D, Ballester J (2019). Trends in temperature-related age-specific and sex-specific mortality from cardiovascular diseases in Spain: a national time-series analysis. The Lancet Planetary Health.

[23] Roger D (2006). Peng FDaTAL: Model choice in time series studies of air pollution and mortality. J R Stat Soc A Stat Soc.

[24] GENTLEMAN NSaJF: On judging the significance of differences by examining the overlap between confidence intervals. Am Stat. 2001;55(3):182–6.

[25] Breitner S, Wolf K, Devlin RB, Diaz-Sanchez D, Peters A, Schneider A (2014). Short-term effects of air temperature on mortality and effect modification by air pollution in three cities of Bavaria, Germany: a time-series analysis. Sci Total Environ.

[26] Mari-Dell'Olmo M, Tobias A, Gomez-Gutierrez A, Rodriguez-Sanz M (2019). Garcia de Olalla P, Camprubi E, Gasparrini A, Borrell C: Social inequalities in the association between temperature and mortality in a South European context. Int J Public Health.

[27] Deng J, Hu X, Xiao C, Xu S, Gao X, Ma Y, Yang J, Wu M, Liu X, Ni J (2020). Ambient temperature and non-accidental mortality: a time series study. Environ Sci Pollut Res Int.

[28] Seposo XT, Dang TN, Honda Y (2015). Evaluating the Effects of Temperature on Mortality in Manila City (Philippines) from 2006–2010 Using a Distributed Lag Nonlinear Model. Int J Environ Res Public Health.

[29] Son JY, Lee JT, Anderson GB, Bell ML (2011). Vulnerability to temperature-related mortality in Seoul, Korea. Environ Res Lett.

[30] Zhang Y, Li S, Pan X, Tong S, Jaakkola J, Gasparrini A, Guo Y (2014). S W: The effects of ambient temperature on cerebrovascular mortality an epidemiologic study in four climatic zones in China. Environ Health Perspect.

[31] Wu W, Xiao Y, Li G, Zeng W, Lin H, Rutherford S, Xu Y, Luo Y, Xu X, Chu C (2013). Temperature-mortality relationship in four subtropical Chinese cities: a time-series study using a distributed lag non-linear model. Sci Total Environ.

[32] Yi W, Chan AP (2015). Effects of temperature on mortality in Hong Kong: a time series analysis. Int J Biometeorol.

[33] Denpetkul T, Phosri A (2021). Daily ambient temperature and mortality in Thailand: Estimated effects, attributable risks, and effect modifications by greenness. Sci Total Environ.

[34] Shindell D, Zhang Y, Scott M, Ru M, Stark K, Ebi KL (2020). The Effects of Heat Exposure on Human Mortality Throughout the United States. Geohealth.

[35] Li J, Xu X, Yang J, Liu Z, Xu L, Gao J, Liu X, Wu H, Wang J, Yu J (2017). Ambient high temperature and mortality in Jinan, China: A study of heat thresholds and vulnerable populations. Environ Res.

[36] Zhang Y, Li C, Feng R, Zhu Y, Wu K, Tan X, Ma L (2016). The Short-Term Effect of Ambient Temperature on Mortality in Wuhan, China: A Time-Series Study Using a Distributed Lag Non-Linear Model. Int J Environ Res Public Health.

[37] Basu R, Ostro BD (2008). A multicounty analysis identifying the populations vulnerable to mortality associated with high ambient temperature in California. Am J Epidemiol.

[38] Lin YK, Ho TJ, Wang YC (2011). Mortality risk associated with temperature and prolonged temperature extremes in elderly populations in Taiwan. Environ Res.

[39] Alahmad B, Shakarchi AF, Khraishah H, Alseaidan M, Gasana J, Al-Hemoud A, Koutrakis P, Fox MA (2020). Extreme temperatures and mortality in Kuwait: Who is vulnerable?. Sci Total Environ.

[40] Iniguez C, Ballester F, Ferrandiz J, Perez-Hoyos S, Saez M, Lopez A, Tempro E (2010). Relation between temperature and mortality in thirteen Spanish cities. Int J Environ Res Public Health.

[41] Kenny GP, Yardley J, Brown C, Sigal RJ, Jay O (2010). Heat stress in older individuals and patients with common chronic diseases. CMAJ.

[42] Wang C, Zhang Z, Zhou M, Zhang L, Yin P, Ye W, Chen Y (2017). Nonlinear relationship between extreme temperature and mortality in different temperature zones: A systematic study of 122 communities across the mainland of China. Sci Total Environ.

[43] Lu P, Zhao Q, Xia G, Xu R, Hanna L, Jiang J, Li S, Guo Y: Temporal trends of the association between ambient temperature and cardiovascular mortality: a 17-year case-crossover study. Environ Res Lett. 2021;16(4).

[44] Han J, Liu S, Zhang J, Zhou L, Fang Q, Zhang J, Zhang Y (2017). The impact of temperature extremes on mortality: a time-series study in Jinan, China. BMJ Open.

[45] Guo Y (2012). KPaST: Effects of temperature on mortality in Chiang Mai city, Thailand a time series study. Environ Health.

[46] Fouillet A, Rey G, Laurent F, Pavillon G, Bellec S, Guihenneuc-Jouyaux C, Clavel J, Jougla E, Hemon D (2006). Excess mortality related to the August 2003 heat wave in France. Int Arch Occup Environ Health.

[47] Vaneckova P, Beggs PJ, Jacobson CR (2010). Spatial analysis of heat-related mortality among the elderly between 1993 and 2004 in Sydney. Australia Soc Sci Med.

[48] Ma W, Yang C, Tan J, Song W, Chen B, Kan H (2012). Modifiers of the temperature-mortality association in Shanghai. China Int J Biometeorol.

[49] Huang W, Kan H, Kovats S (2010). The impact of the 2003 heat wave on mortality in Shanghai. China Sci Total Environ.

[50] Onozuka D, Hagihara A (2015). Variation in vulnerability to extreme-temperature-related mortality in Japan: A 40-year time-series analysis. Environ Res.

[51] Yang J (2012). C-QO, Yan Ding, Y-XZaP-YC: Daily temperature and mortality a study of distributed lag non-linear effect and effect modification in Guangzhou. Environ Health.

[52] Medina-Ramon M, Zanobetti A, Cavanagh DP, Schwartz J (2006). Extreme temperatures and mortality: assessing effect modification by personal characteristics and specific cause of death in a multi-city case-only analysis. Environ Health Perspect.

[53] Mirabelli MAMaM: The potential impacts of climate variability and change on temperature-related morbidity and mortality in the United States. Environ Health Perspect. 2001;109(suppl 2):185–9. 10.1289/ehp.109-1240665 PMC1240665 11359685

[54] Son JY, Gouveia N, Bravo MA, de Freitas CU, Bell ML (2016). The impact of temperature on mortality in a subtropical city: effects of cold, heat, and heat waves in Sao Paulo. Brazil Int J Biometeorol.

[55] Robards J, Evandrou M, Falkingham J, Vlachantoni A (2012). Marital status, health and mortality. Maturitas.

[56] Goldman YHaN (1990). Mortality differentials by marital status an international comparison. Demography.

[57] NOREEN GOLDMAN SKaRW (1995). Marital status and health among the elderly. Soc Sci Med.

[58] Eaker ED, Sullivan LM, Kelly-Hayes M, D'Agostino RB, Benjamin EJ (2007). Marital status, marital strain, and risk of coronary heart disease or total mortality: the Framingham Offspring Study. Psychosom Med.

[59] Yin Q, Wang J (2017). The association between consecutive days' heat wave and cardiovascular disease mortality in Beijing, China. BMC Public Health.

[60] Karpov VY, Zavalishina SY, Bakulina ED, Dorontsev AV, Gusev AV, Fedorova TY, Okolelova VA (2021). The Physiological Response of the Body to Low Temperatures. J Biochem Technol.

[61] Degroot DW, Kenney WL (2007). Impaired defense of core temperature in aged humans during mild cold stress. Am J Physiol Regul Integr Comp Physiol.

