# Supplementary material for: Effects of ambient temperature on mortality among elderly residents of Chengdu city in Southwest China, 2016–2020: a distributed-lag non-linear time series analysis
Source: BMC Public Health. 2023 Jan 21;23:149. doi: 10.1186/s12889-022-14931-x (PMC9863161; doi:10.1186/s12889-022-14931-x)
Supplement: Supplementary file 1 — Additional file 1: Table S1. Time trend freedom 6-9 Table S2. Air pollutants and relative humidity trend freedom 3-5. [file 12889_2022_14931_MOESM1_ESM.docx]

**Table S1 Time trend freedom 6-9**

| **Diseases** | **RR(cold)** | **RR(hot)** | **RR 95% CI**(cold) | | **RR 95% CI**(hot) | | **Lags (day)** | **DFs** |
| --- | --- | --- | --- | --- | --- | --- | --- | --- |
|  |  |  | **Lower** | **Upper** | **Lower** | **Upper** |  |  |
| **Total** | 1.001 | **1.065*** | 0.987 | 1.016 | 1.049 | 1.080 | 1 | 6 |
|  | **1.033*** | 1.002 | 1.022 | 1.045 | 0.992 | 1.012 | 3 | 6 |
|  | **1.027*** | 0.979 | 1.018 | 1.037 | 0.972 | 0.987 | 7 | 6 |
|  | **1.029*** | 0.976 | 1.003 | 1.055 | 0.953 | 1.000 | 14 | 6 |
|  | 0.995 | **1.066*** | 0.979 | 1.011 | 1.052 | 1.081 | 1 | 7 |
|  | **1.035*** | 1.007 | 1.022 | 1.047 | 0.998 | 1.017 | 3 | 7 |
|  | **1.030*** | 0.985 | 1.020 | 1.041 | 0.977 | 0.992 | 7 | 7 |
|  | **1.029*** | 0.979 | 1.001 | 1.058 | 0.958 | 1.002 | 14 | 7 |
|  | 0.991 | **1.063*** | 0.975 | 1.007 | 1.049 | 1.077 | 1 | 8 |
|  | 1.036 | 1.009 | 1.023 | 1.049 | 0.999 | 1.018 | 3 | 8 |
|  | **1.034*** | 0.988 | 1.024 | 1.045 | 0.980 | 0.995 | 7 | 8 |
|  | **1.033*** | 0.986 | 1.004 | 1.063 | 0.965 | 1.007 | 14 | 8 |
|  | 0.992 | **1.063*** | 0.976 | 1.009 | 1.050 | 1.077 | 1 | 9 |
|  | **1.038*** | **1.009*** | 1.025 | 1.051 | 1.000 | 1.019 | 3 | 9 |
|  | **1.036*** | 0.988 | 1.025 | 1.047 | 0.981 | 0.995 | 7 | 9 |
|  | **1.034*** | 0.990 | 1.005 | 1.064 | 0.969 | 1.011 | 14 | 9 |
| **CVD** | 1.016 | **1.103*** | 0.993 | 1.040 | 1.080 | 1.126 | 1 | 6 |
|  | **1.028*** | 1.009 | 1.011 | 1.047 | 0.994 | 1.025 | 3 | 6 |
|  | **1.033*** | 0.974 | 1.018 | 1.048 | 0.963 | 0.986 | 7 | 6 |
|  | 1.012 | 0.976 | 0.973 | 1.054 | 0.941 | 1.012 | 14 | 6 |
|  | 1.015 | **1.109** ***** | 0.992 | 1.039 | 1.085 | 1.133 | 1 | 7 |
|  | **1.028*** | 1.013 | 1.010 | 1.047 | 0.998 | 1.028 | 3 | 7 |
|  | **1.033*** | 0.977 | 1.018 | 1.048 | 0.966 | 0.989 | 7 | 7 |
|  | 1.008 | 0.977 | 0.968 | 1.050 | 0.943 | 1.012 | 14 | 7 |
|  | 1.010 | **1.106 *** | 0.985 | 1.035 | 1.084 | 1.129 | 1 | 8 |
|  | **1.029*** | 1.014 | 1.010 | 1.049 | 0.999 | 1.029 | 3 | 8 |
|  | **1.037*** | 0.980 | 1.021 | 1.053 | 0.969 | 0.991 | 7 | 8 |
|  | 1.010 | 0.981 | 0.968 | 1.054 | 0.948 | 1.015 | 14 | 8 |
|  | 1.008 | **1.102*** | 0.983 | 1.034 | 1.081 | 1.124 | 1 | 9 |
|  | **1.030*** | 1.014 | 1.010 | 1.050 | 0.999 | 1.028 | 3 | 9 |
|  | **1.040*** | 0.982 | 1.023 | 1.056 | 0.971 | 0.993 | 7 | 9 |
|  | 1.010 | 0.987 | 0.967 | 1.055 | 0.954 | 1.021 | 14 | 9 |
| **Resp** | 1.000 | **1.098*** | 0.981 | 1.018 | 1.067 | 1.130 | 1 | 6 |
|  | **1.034*** | 1.009 | 1.019 | 1.049 | 0.989 | 1.030 | 3 | 6 |
|  | **1.025*** | 0.979 | 1.013 | 1.036 | 0.964 | 0.995 | 7 | 6 |
|  | **1.045*** | 0.970 | 1.011 | 1.080 | 0.925 | 1.017 | 14 | 6 |
|  | 0.987 | **1.099*** | 0.966 | 1.009 | 1.071 | 1.128 | 1 | 7 |
|  | **1.038*** | **1.019*** | 1.021 | 1.056 | 1.001 | 1.037 | 3 | 7 |
|  | **1.026*** | 0.983 | 1.012 | 1.040 | 0.970 | 0.997 | 7 | 7 |
|  | **1.048*** | 0.974 | 1.008 | 1.090 | 0.933 | 1.016 | 14 | 7 |
|  | 0.979 | **1.090*** | 0.954 | 1.004 | 1.063 | 1.116 | 1 | 8 |
|  | **1.044*** | **1.023*** | 1.024 | 1.064 | 1.005 | 1.041 | 3 | 8 |
|  | **1.032*** | 0.988 | 1.016 | 1.049 | 0.974 | 1.001 | 7 | 8 |
|  | **1.062*** | 0.989 | 1.016 | 1.110 | 0.950 | 1.030 | 14 | 8 |
|  | 0.979 | **1.088*** | 0.954 | 1.004 | 1.063 | 1.114 | 1 | 9 |
|  | **1.047*** | **1.025*** | 1.026 | 1.068 | 1.008 | 1.043 | 3 | 9 |
|  | **1.036*** | 0.991 | 1.019 | 1.053 | 0.978 | 1.004 | 7 | 9 |
|  | **1.066*** | 0.999 | 1.019 | 1.114 | 0.960 | 1.040 | 14 | 9 |
| **cere** | 1.029 | **1.070*** | 0.993 | 1.066 | 1.042 | 1.100 | 1 | 6 |
|  | 1.023 | 1.001 | 0.996 | 1.051 | 0.981 | 1.020 | 3 | 6 |
|  | **1.032*** | 0.983 | 1.010 | 1.055 | 0.968 | 0.998 | 7 | 6 |
|  | 0.979 | 0.961 | 0.920 | 1.041 | 0.916 | 1.007 | 14 | 6 |
|  | 1.031 | **1.080*** | 0.995 | 1.068 | 1.049 | 1.112 | 1 | 7 |
|  | 1.022 | 1.005 | 0.995 | 1.049 | 0.985 | 1.026 | 3 | 7 |
|  | **1.029*** | 0.984 | 1.007 | 1.052 | 0.969 | 1.000 | 7 | 7 |
|  | 0.971 | 0.961 | 0.914 | 1.032 | 0.916 | 1.008 | 14 | 7 |
|  | 1.031 | **1.087*** | 0.995 | 1.068 | 1.055 | 1.120 | 1 | 8 |
|  | 1.022 | 1.009 | 0.994 | 1.050 | 0.988 | 1.031 | 3 | 8 |
|  | **1.030*** | 0.988 | 1.008 | 1.053 | 0.972 | 1.004 | 7 | 8 |
|  | 0.970 | 0.971 | 0.913 | 1.032 | 0.924 | 1.019 | 14 | 8 |
|  | 1.031 | **1.080*** | 0.994 | 1.070 | 1.051 | 1.110 | 1 | 9 |
|  | 1.025 | 1.008 | 0.996 | 1.054 | 0.989 | 1.028 | 3 | 9 |
|  | **1.035*** | 0.989 | 1.012 | 1.059 | 0.974 | 1.005 | 7 | 9 |
|  | 0.973 | 0.978 | 0.912 | 1.037 | 0.934 | 1.025 | 14 | 9 |
| **IHD** | 0.991 | **1.112*** | 0.955 | 1.028 | 1.064 | 1.162 | 1 | 6 |
|  | 1.020 | 1.019 | 0.991 | 1.049 | 0.988 | 1.051 | 3 | 6 |
|  | 1.012 | 0.965 | 0.989 | 1.035 | 0.942 | 0.989 | 7 | 6 |
|  | **1.071*** | 1.013 | 1.003 | 1.144 | 0.942 | 1.091 | 14 | 6 |
|  | 0.985 | **1.117*** | 0.944 | 1.028 | 1.070 | 1.165 | 1 | 7 |
|  | 1.021 | 1.025 | 0.988 | 1.055 | 0.996 | 1.056 | 3 | 7 |
|  | 1.014 | 0.971 | 0.988 | 1.041 | 0.949 | 0.993 | 7 | 7 |
|  | 1.067 | 1.014 | 0.990 | 1.150 | 0.946 | 1.086 | 14 | 7 |
|  | 0.981 | **1.108*** | 0.938 | 1.026 | 1.063 | 1.155 | 1 | 8 |
|  | 1.024 | 1.023 | 0.989 | 1.059 | 0.993 | 1.053 | 3 | 8 |
|  | 1.019 | 0.971 | 0.991 | 1.048 | 0.949 | 0.994 | 7 | 8 |
|  | 1.074 | 1.013 | 0.993 | 1.161 | 0.946 | 1.084 | 14 | 8 |
|  | 0.978 | **1.102*** | 0.933 | 1.026 | 1.059 | 1.146 | 1 | 9 |
|  | 1.028 | 1.019 | 0.992 | 1.066 | 0.991 | 1.049 | 3 | 9 |
|  | 1.026 | 0.971 | 0.996 | 1.057 | 0.950 | 0.993 | 7 | 9 |
|  | 1.084 | 1.007 | 0.999 | 1.176 | 0.942 | 1.076 | 14 | 9 |

RR, relative risk; CI, confidence interval.

^*^*P* < 0.05.

**Table S2 Air pollutants and relative humidity trend freedom 3-5**

| **Diseases** | **RR(cold)** | **RR(hot)** | **RR 95% CI**(cold) | | **RR 95% CI**(hot) | | **Lags (day)** | **DFs** |
| --- | --- | --- | --- | --- | --- | --- | --- | --- |
|  |  |  | **Lower** | **Upper** | **Lower** | **Upper** |  |  |
| **Total** | 0.991 | **1.063*** | 0.975 | 1.007 | 1.049 | 1.077 | 1 | 3 |
|  | **1.036*** | 1.009 | 1.023 | 1.049 | 0.999 | 1.018 | 3 | 3 |
|  | **1.034*** | 0.988 | 1.024 | 1.045 | 0.980 | 0.995 | 7 | 3 |
|  | **1.033*** | 0.986 | 1.004 | 1.063 | 0.965 | 1.007 | 14 | 3 |
|  | 0.991 | **1.063*** | 0.975 | 1.008 | 1.049 | 1.077 | 1 | 4 |
|  | **1.037*** | 1.009 | 1.024 | 1.050 | 0.999 | 1.018 | 3 | 4 |
|  | **1.034*** | 0.988 | 1.024 | 1.045 | 0.980 | 0.995 | 7 | 4 |
|  | **1.033*** | 0.986 | 1.004 | 1.063 | 0.965 | 1.007 | 14 | 4 |
|  | 0.991 | **1.064*** | 0.975 | 1.008 | 1.050 | 1.078 | 1 | 5 |
|  | **1.037*** | **1.009*** | 1.024 | 1.050 | 1.000 | 1.019 | 3 | 5 |
|  | **1.034*** | 0.987 | 1.024 | 1.045 | 0.980 | 0.995 | 7 | 5 |
|  | **1.033*** | 0.986 | 1.004 | 1.063 | 0.965 | 1.007 | 14 | 5 |
| **CVD** | 1.010 | **1.106*** | 0.985 | 1.035 | 1.084 | 1.129 | 1 | 3 |
|  | **1.029*** | 1.014 | 1.010 | 1.049 | 0.999 | 1.029 | 3 | 3 |
|  | **1.037*** | 0.980 | 1.021 | 1.053 | 0.969 | 0.991 | 7 | 3 |
|  | 1.010 | 0.981 | 0.968 | 1.054 | 0.948 | 1.015 | 14 | 3 |
|  | 1.010 | **1.107*** | 0.985 | 1.035 | 1.084 | 1.130 | 1 | 4 |
|  | **1.029*** | 1.014 | 1.010 | 1.049 | 0.999 | 1.029 | 3 | 4 |
|  | **1.038*** | 0.980 | 1.022 | 1.053 | 0.969 | 0.991 | 7 | 4 |
|  | 1.009 | 0.981 | 0.967 | 1.053 | 0.948 | 1.015 | 14 | 4 |
|  | 1.011 | **1.108*** | 0.986 | 1.036 | 1.085 | 1.131 | 1 | 5 |
|  | **1.029*** | **1.015*** | 1.010 | 1.049 | 1.000 | 1.031 | 3 | 5 |
|  | **1.038*** | 0.980 | 1.022 | 1.054 | 0.969 | 0.991 | 7 | 5 |
|  | 1.007 | 0.983 | 0.965 | 1.051 | 0.950 | 1.018 | 14 | 5 |
| **Resp** | 0.979 | **1.090*** | 0.954 | 1.004 | 1.063 | 1.116 | 1 | 3 |
|  | **1.044*** | **1.023*** | 1.024 | 1.064 | 1.005 | 1.041 | 3 | 3 |
|  | **1.032*** | 0.988 | 1.016 | 1.049 | 0.974 | 1.001 | 7 | 3 |
|  | **1.062*** | 0.989 | 1.016 | 1.110 | 0.950 | 1.030 | 14 | 3 |
|  | 0.979 | **1.089*** | 0.955 | 1.004 | 1.063 | 1.116 | 1 | 4 |
|  | **1.044*** | **1.023*** | 1.024 | 1.064 | 1.005 | 1.041 | 3 | 4 |
|  | **1.031*** | 0.988 | 1.015 | 1.048 | 0.974 | 1.001 | 7 | 4 |
|  | **1.063*** | 0.989 | 1.017 | 1.110 | 0.949 | 1.030 | 14 | 4 |
|  | 0.980 | **1.090*** | 0.955 | 1.005 | 1.063 | 1.117 | 1 | 5 |
|  | **1.044*** | **1.023*** | 1.024 | 1.065 | 1.005 | 1.041 | 3 | 5 |
|  | **1.031*** | 0.988 | 1.015 | 1.048 | 0.974 | 1.001 | 7 | 5 |
|  | **1.063*** | 0.989 | 1.017 | 1.111 | 0.95 | 1.030 | 14 | 5 |
| **cere** | 1.031 | **1.087*** | 0.995 | 1.068 | 1.055 | 1.120 | 1 | 3 |
|  | 1.022 | 1.009 | 0.994 | 1.050 | 0.988 | 1.031 | 3 | 3 |
|  | **1.030*** | 0.988 | 1.008 | 1.053 | 0.972 | 1.004 | 7 | 3 |
|  | 0.970 | 0.971 | 0.913 | 1.032 | 0.924 | 1.019 | 14 | 3 |
|  | 1.029 | **1.086*** | 0.993 | 1.066 | 1.054 | 1.118 | 1 | 4 |
|  | 1.022 | 1.009 | 0.994 | 1.050 | 0.988 | 1.030 | 3 | 4 |
|  | **1.031*** | 0.988 | 1.008 | 1.054 | 0.972 | 1.004 | 7 | 4 |
|  | 0.972 | 0.972 | 0.914 | 1.034 | 0.926 | 1.021 | 14 | 4 |
|  | 1.031 | **1.087*** | 0.995 | 1.068 | 1.055 | 1.120 | 1 | 5 |
|  | 1.022 | 1.011 | 0.994 | 1.050 | 0.990 | 1.032 | 3 | 5 |
|  | **1.030*** | 0.988 | 1.008 | 1.053 | 0.972 | 1.005 | 7 | 5 |
|  | 0.970 | 0.975 | 0.912 | 1.032 | 0.928 | 1.024 | 14 | 5 |
| **IHD** | 0.981 | **1.108*** | 0.938 | 1.026 | 1.063 | 1.155 | 1 | 3 |
|  | 1.024 | 1.023 | 0.989 | 1.059 | 0.993 | 1.053 | 3 | 3 |
|  | 1.019 | 0.971 | 0.991 | 1.048 | 0.949 | 0.994 | 7 | 3 |
|  | 1.074 | 1.013 | 0.993 | 1.161 | 0.946 | 1.084 | 14 | 3 |
|  | 0.981 | **1.111*** | 0.938 | 1.026 | 1.065 | 1.158 | 1 | 4 |
|  | 1.024 | 1.023 | 0.989 | 1.059 | 0.993 | 1.053 | 3 | 4 |
|  | 1.021 | 0.970 | 0.992 | 1.050 | 0.948 | 0.993 | 7 | 4 |
|  | 1.070 | 1.012 | 0.989 | 1.157 | 0.946 | 1.084 | 14 | 4 |
|  | 0.981 | **1.113*** | 0.938 | 1.026 | 1.067 | 1.160 | 1 | 5 |
|  | 1.024 | 1.024 | 0.990 | 1.060 | 0.994 | 1.055 | 3 | 5 |
|  | 1.021 | 0.970 | 0.993 | 1.050 | 0.948 | 0.993 | 7 | 5 |
|  | 1.070 | 1.013 | 0.989 | 1.158 | 0.946 | 1.085 | 14 | 5 |

RR, relative risk; CI, confidence interval.

^*^*P* < 0.05.
